# The cannabinoid quinol VCE-004.8 alleviates bleomycin-induced scleroderma and exerts potent antifibrotic effects through peroxisome proliferator-activated receptor-γ and CB2 pathways

**DOI:** 10.1038/srep21703

**Published:** 2016-02-18

**Authors:** Carmen del Río, Carmen Navarrete, Juan A. Collado, M. Luz Bellido, María Gómez-Cañas, M. Ruth Pazos, Javier Fernández-Ruiz, Federica Pollastro, Giovanni Appendino, Marco A. Calzado, Irene Cantarero, Eduardo Muñoz

**Affiliations:** 1 Maimonides Biomedical Research Institute of Córdoba, Reina Sofía University Hospital, Dept of Cell Biology, Physiology and Immunology, University of Córdoba, Avda Menéndez Pidal s/n, 14004. Córdoba, Spain; 2 VivaCell Biotechnology España, Parque Científico Tecnológico Rabanales 21, 14014, Córdoba, Spain; 3 Instituto Universitario de Investigación en Neuroquímica, Departamento de Bioquímica y Biología Molecular III, Facultad de Medicina, Universidad Complutense, Madrid, Spain; 4 Centro de Investigación Biomédica en Red sobre Enfermedades Neurodegenerativas (CIBERNED), Madrid, Spain; 5 Instituto Ramón y Cajal de Investigación Sanitaria (IRYCIS), Madrid, Spain; 6 Dipartimento di Scienze Chimiche, Alimentari, Farmaceutiche e Farmacologiche, Via Bovio 6, 28100, Novara, Italy

## Abstract

Scleroderma is a group of rare diseases associated with early and transient inflammation and vascular injury, followed by fibrosis affecting the skin and multiple internal organs. Fibroblast activation is the hallmark of scleroderma, and disrupting the intracellular TGFβ signaling may provide a novel approach to controlling fibrosis. Because of its potential role in modulating inflammatory and fibrotic responses, both PPARγ and CB_2_ receptors represent attractive targets for the development of cannabinoid-based therapies. We have developed a non-thiophilic and chemically stable derivative of the CBD quinol (VCE-004.8) that behaves as a dual agonist of PPARγ and CB_2_ receptors, VCE-004.8 inhibited TGFβ-induced Col1A2 gene transcription and collagen synthesis. Moreover, VCE-004.8 inhibited TGFβ–mediated myofibroblast differentiation and impaired wound-healing activity. The anti-fibrotic efficacy *in vivo* was investigated in a murine model of dermal fibrosis induced by bleomycin. VCE-004.8 reduced dermal thickness, blood vessels collagen accumulation and prevented mast cell degranulation and macrophage infiltration in the skin. These effects were impaired by the PPARγ antagonist T0070907 and the CB_2_ antagonist AM630. In addition, VCE-004.8 downregulated the expression of several key genes associated with fibrosis, qualifying this semi-synthetic cannabinoid as a novel compound for the management of scleroderma and, potentially, other fibrotic diseases.

Systemic sclerosis (SSc; scleroderma) is a rare and heterogeneous disease that involves three main hallmarks: fibroblast dysfunction leading to increased deposition of extracellular matrix proteins, small vessel vasculopathy resulting in tissue hypoxia and an immune response with autoantibody production^1^. SSc is characterized by progressive thickening and fibrosis of skin secondary to excessive collagen accumulation, that can be limited to the skin (limited cutaneous SSc) or extended to internal organs (diffuse cutaneous SSc)^2^.

SSc is initiated by microvascular injury and inflammation followed by fibroblast activation, a key event in fibrosis development. Activated fibroblasts are more resistant to apoptosis and differentiate to myofibroblasts, which are responsible for the excessive collagen synthesis and Transforming Growth Factor beta (TGFβ) production^3^. Following TGFβ binding to its receptors, SMAD2 and SMAD3 are phosphorylated and associated with SMAD4. The resulting heteromultimer translocates to the nucleus, where it regulates the expression of TGFβ target genes like collagen type I^4^. Excessive TGFβ signaling is the hallmark of SSc and different strategies aimed to disrupt the TGFβ/SMAD signaling pathway have been proposed for the treatment of SSc and related fibrotic diseases^5^. Different studies prove that peroxisome proliferator-activated receptor-γ (PPARγ) as well as cannabinoid receptor type-2 (CB_2_) are potential therapeutic targets for the disease because of their involvement in the inhibition of inflammation and fibrosis progression.

PPARγ is a nuclear receptor originally implicated in the regulation of cell growth, lipid metabolism and glucose homeostasis and it is a target for some antidiabetic drugs^6^. However, PPARγ is broadly expressed and has been recognized to play a key role in inflammatory processes and connective tissue homeostasis^7^. In this sense, loss of PPARγ function in fibroblasts resulted in increased susceptibility to skin fibrosis in mice. Conversely, PPARγ agonists such as rosiglitazone (RGZ) have shown to prevent inflammation, dermal fibrosis and lipoatrophy in a mouse model of cutaneous sclerosis^8^. Moreover, it is known that PPARγ expression and function are impaired in scleroderma by different factors (TGFβ, CTGF, IFNγ, Wnt proteins, IL-13, hypoxia) while fibrosis development is associated to a mutual inhibitory interaction between TGFβ and PPARγ that contributes to disease progression^9^,^10^. It has also been shown that PPARγ agonists inhibit the profibrotic response through inhibition of the TGFβ/SMADs signaling pathway at the nuclear level^10^,^11^.

In addition to antidiabetic drugs, PPARγ is also activated by some endocannabinoids and related signaling lipids, as well as by certain natural and synthetic cannabinoids^12^,^13^,^14^. Cannabinoids are a complex group of molecules that comprise phytocannabinoids, endocannabinoids and synthetic cannabinoids. Cannabinoids were initially identified by their ability to bind and activate the classical cannabinoid receptors CB_1_ and CB_2_, but these compounds are pleiotropic in nature and also activate other type of receptors including PPARγ^12^. Thus, the multitarget activity of cannabinoids may account for their ability to modulate several key processes including neuroprotection, inflammation, immunomodulation and vascular responses^15^,^16^.

Recent evidence indicates that genetic and pharmacological manipulation of the endocannabinoid system modulates the fibrotic response. Thus, CB_1_ and CB_2_ receptors have shown different patterns in experimental models of dermal fibrosis. CB_1_ inactivation prevents fibroblast activation and exerts potent antifibrotic effect^17^. The role of CB_1_ as a pro-fibrotic receptor has also been confirmed in fatty acid amide hydrolase knock-out mice, in which elevated levels of endocannabinoids may induce fibrosis in a CB_1_-dependent manner^18^. In contrast, CB_2_ activation protected mice from experimental dermal fibrosis and tissue leukocyte infiltration^19^. Accordingly, replace by WIN-55,212-2 in all the manuscript (highlighted), a synthetic dual CB_1_/CB_2_ agonist, prevented bleomycin-induced dermal fibrosis^20^, and ajulemic acid, a dual PPARγ/CB_2_ agonist, was also antifibrotic in the same mouse model of SSc^14^. Finally, JWH-133, a selective CB_2_ agonist, was found to alleviate diffuse SSc induced by hypochlorite injections and CB_2_ receptors deficient mice were more susceptible to fibrosis development in this model^21^.

Considering that dual PPARγ/CB_2_ agonists have a strong potential as disease-modifying agents in SSc, we have investigated the novel non-psychotropic cannabidiol (CBD) quinol derivative VCE-004.8 for its activity at both targets and its capacity to prevent fibrosis in experimental models of SSc. VCE-004.8 is a chemically stable and non-thiophilic analogue of HU-331, the major product of atmospheric oxidation of CBD in basic medium.

## Material and Methods

### Cell lines and reagents

NIH-3T3, HEK-293T-CB_2_, normal human dermal fibroblasts (NHDFs) and mouse embryonic fibroblasts (MEFs) cells were cultured in DMEM supplemented with 10% fetal bovine serum (FBS), 2 mM L-glutamine and 1% (v/v) penicillin/streptomycin and maintained at 37 °C in a humidified atmosphere containing 5% CO_2_. Rosiglitazone, AM630, T0070907 and WIN-55,212-2 were obtained from Cayman Chem (MI, USA), TGFβ1 was purchased from Immunotools GmbH (Friesoythe, Germany), CBD was purchased to THC Pharm (Frankfurt, Germany) and HU-331 was from Santa Cruz (CA, USA). All other reagents were from Sigma Co (St Louis, MO, USA). The synthesis and structure of VCE-004.8 is described in supplementary information.

### PPARγ, CB_1_ and CB_2_ binding assays

To determine PPARγ binding activity the PolarScreen^TM^ PPARY Competitor Assay kit (Life Technologies, CA, USA) was used following manufacturer’s instructions. The IC_50_ value was determined as the 50% inhibition of percentage of polarization. CB_1_ and CB_2_ receptor binding affinities were determined by competition studies using [^3^H]CP55940 as radioligand and commercial membrane preparations of HEK293 EBNA cells stably expressing the respective receptor type (Perkin-Elmer Life and Analytical Sciences, Boston, MA), following a procedure previously described^22^. Initially, compounds were screened at 40 μM and, in those cases in which the inhibition of radioligand binding was at least about 70%, competition curves using a broad range of compound concentrations were performed to calculate Ki values. Competition binding data were analyzed by using GraphPad Prism^®^ version 5.01 (GraphPad Software Inc., CA, USA).

### Transient transfections and luciferase reporter assays

The cells were seeded in 24-well plates and after 24 hours they were transiently transfected with the indicated constructs using Roti^©^-Fect (Carl Roth, Karlsruhe, Germany) following manufacturer´s specifications. After stimulation, the luciferase activities were quantified using Dual-Luciferase Assay (Promega, Madison, WI, USA). To correct for transfection efficacy, 100 ng *Renilla* luciferase (pRL-CMV) was cotransfected.

### Cytotoxicity assays

Briefly, NIH-3T3 cells were seeded at a density of 1 × 10^3 ^cells/well in 96-well plates and treated with increasing concentrations of VCE-004.8 or HU-331 for 24 hours. After that, 50 μl of 3-MTT (4,5-Dimethylthiazol-2-yl)-2,5-diphenyltetrazolium bromide) (5 mg/ml) from a mixture solution of MTT: DMEM (1:2) per well was added and cells were incubated for 4 hours at 37  °C in darkness. Then, supernatant was removed and 100 μl DMSO was added to each well for 10 min, in gentle shaking. Absorbance was measured at 550 nm using a TriStar LB 941 (Berthold Technologies, GmbH & Co. KG). The mean concentration in each set of three wells was measured. The absorbance of untreated controls was considered as 100% survival.

### Determination of collagen in NHDFs

To determine collagen deposition, NHDFs cells were seeded in 24-well plates (5 × 10^4^/well), preincubated with VCE-004.8 for 1 hour and stimulated with TGFβ1 (5 ng/ml) during 48 hours. Cell pellets were extracted with 0.5 M acetic acid and dyed for 30 min (0,1% Sirius Red and 0,1% Fast Green dissolved in saturated picric acid). Collagen deposition was measured at 540 nm and 605 nm in Genesis 10 UV spectrofluorometer (Thermo Fisher Scientific). To study the secretion of collagen, NHDFs cells were seeded in 24-well plates (5 × 10^4^/well) and cultivated in serum-free medium for 24 h. After that, the cells were preincubated with VCE-004.8 for 1 hour and stimulated with TGFβ1 (10 ng/ml) for 48 h in serum-free medium and the supernatants collected and tested using the Procollagen Type I C-Peptide (PIP) EIA Kit (Takara, Japan) following manufacturer’s recommendations.

### Miofibroblast differentiation

To induce myofibroblast differentiation MEFs were plated on glass coverslips with the appropriate medium. After serum starvation for 24 h, the cells were preincubated with RGZ or VCE-004.8 for 1 h and stimulated with TGFβ1 (10 ng/ml) for another 24 h. Coverslips were collected, washed with PBS and fixed with 4% paraformaldehyde. After antigen retrieval non-specific binding was reduced by blocking with IHC select blocking reagent (Merck Millipore, MA, USA) at room temperature for 30 min. Coverslips were placed in a humidified chamber and incubated overnight at 4 °C with a primary antibody α-SMA (1:50, sc-32251, Santa Cruz). Then, coverslips were washed three times with 0.1% PBS-Tween 20 and incubated with the secondary antibody goat anti-rat Alexa 488 (Merck Millipore). Finally, coverslips were mounted in VectaShield Mounting Medium with DAPI and analyzed using a Leica DM2500 microscope and a Leica DFC420c camera.

### Western blots

Cells were washed with PBS and proteins extracted in 50 μl of lysis buffer (50 mM Tris–HCl pH 7.5, 150 mM NaCl, 10% glycerol and 1% NP-40) supplemented with 10 mM NaF, 1 mM Na_3_VO_4_, 10 μg/ml leupeptine, 1 μg/ml pepstatin and aprotinin, and 1 μl/ml PMSF saturated. Protein concentration was determined by the Bradford assay (Bio-Rad, CA, USA) and 30 μg of proteins were boiled at 95 °C in Laemmli buffer and electrophoresed in 10% SDS/PAGE gels. Separated proteins were transferred to PVDF membranes (20V for 30 min) and blocked in TBS solution containing 0.1% Tween 20 and 5% non-fat dry milk for 1 hour at room temperature. Immunodetection of specific proteins was carried out by incubation with primary antibody against α-SMA (1:500; sc-32251, Santa Cruz), pSMAD2 (1:500; AB3849, Merck Millipore) or SMAD2 (1:1000; 5339, Cell Signaling, MA, USA) overnight at 4 °C. After washing membranes, horseradish peroxidase-conjugated secondary antibody was added and detected by chemiluminescence system (GE Healthcare Europe GmbH). The blots were reproved with an antibody against α-tubulin or β-actin (1:10.000; DM-1A and AC-74, Sigma). Densitometric analysis of immunoblots was performed using Image J software (NIH; Bethesda, MD, USA).

### *In vitro* cell migration assays

The modulation of cell migration was analyzed by wound-healing assays. Briefly, NHDFs were seeded in a 96-well Essen ImageLock plate (Essen BioScience) and were grown to confluence. After 24 h, the scratches were made using the 96-pin WoundMaker (Essen BioScience), followed by incubation of the cultures in media with 10 ng/ml of mitomycin C to block cell proliferation. VCE-004.8 and TGFβ1 or rhIL-4 (10 ng/ml) were added and wound images were taken every 60 min for 36 h, and the data analyzed by the integrated metric Relative Wound Density part of the live content cell imaging system IncuCyte HD (Essen BioScience).

### Animals and Experimental protocols

Six- to eight-week-old female BALB/c mice were purchased from Harlan laboratories (Barcelona, Spain) and housed in our animal facilities (University of Córdoba, Córdoba, Spain) under controlled conditions (12 h light/dark cycle; temperature 20 ^o^C (±2 ^o^C) and 40–50% relative humidity) with free access to standard food and water. All experiments were performed in accordance with European Union guideline and approved by the Animal Research Ethic Committee of the Córdoba University (2014PI/016). Dermal fibrosis was induced by daily subcutaneous injections of filter-sterilized bleomycin (20 μg/mouse diluted in PBS) (Mylan, Spain) into the shaved backs well-defined areas (1 cm^2^) of mice for 6 weeks. During the last 3 weeks of bleomycin injections, mice were treated in parallel by daily intraperitoneal injection of RGZ (5 mg/kg) or VCE004.8 (10 and 20 mg/kg). In some experiments, mice received a combination of VCE-004.8 (20 mg/kg) and the CB2 antagonist AM630 (2.5 mg/kg), or the PPARγ antagonist T0070907 (5 mg/kg), injected 15 minutes before VCE-004.8. Control group and VCE-004.8 group received subcutaneous injections of 100 μl PBS and intraperitoneal injections of vehicle or VCE-004.8 (20 mg/kg), respectively. At the end of the experiment, mice were sacrificed and dissected for tissue processing. Affected skin was frozen in RNA-later cooled in dry ice and stored at −80 °C for biochemical analyses (PCR array system) or fixed in fresh 4% paraformaldehyde (PBS) for histochemical analysis. Eight to ten animals were analyzed per experimental group.

### Histochemical analysis

Five-micrometer–thick sections of formalin-fixed paraffin-embedded skin biopsy samples were stained for hematoxylin and eosin, Masson’s trichrome or toluidine blue. Only nuclear cells were counted. For immunohistochemical analysis, antigen retrieval was performed in 37 °C trypsin (pH 7.8) for 1 h. Sections were incubated with F4/80 antibody (1:50; MCA497, Bio-Rad) for 2 hours at room temperature or with p-SMAD2 antibody (1:500) overnight at 4 °C. Then, sections were washed in 0.1 M PBS and incubated for 1 hour at room temperature with the appropriate biotin-conjugated secondary antibody (Merck Millipore). Reaction was revealed with 3,3-diaminobenzidine (DAB) (Merck Millipore). A Leica DM2500 microscope and a Leica DFC420c camera were used for slide observation and photography.

### PCR array

RNeasy Fibrous Tissue Mini kit (Qiagen, Hilden, Germany) was used to isolate total mRNA from the skin. One microgram of RNA was transcribed to single chain cDNA using iScript^TM^ cDNA Synthesis Kit (Bio-Rad) and analyzed using the RT^2^ Profiler^TM^ PCR Array Mouse Fibrosis (Qiagen). This array contains 84 key genes involved in fibrosis development. Data were analyzed using the 2^−ΔΔCt^ method and normalized with five housekeeping genes.

### Statistical analysis

*In vitro* data are expressed as mean ± S.D. and *in vivo* results are represented as mean ± SEM. Data were subjected to Kolmogorov-Smirnov normality test and then, differences were analyzed by one-way ANOVA followed by Tukey post hoc test. When data were not normally distributed, significant differences were studied using the Kruskall-Wallis followed by Dunns post-hoc test. *P* < 0.05 was considered significant. Statistical analysis was performed using GraphPad Prism^®^ version 5.01. Images were analyzed and quantified using the Image J.

## Results

### VCE-004.8 is a stable and non-thiophilic cannabinoid quinol

**Figure 1:**
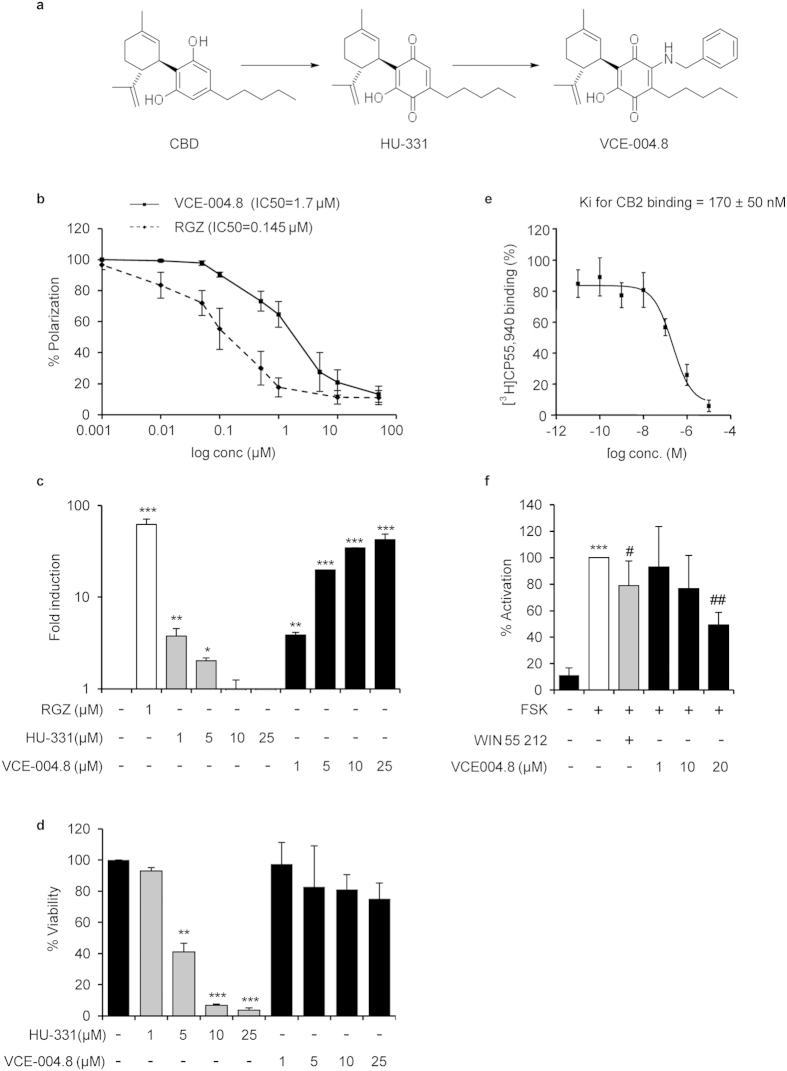
VCE-004.8 characterization. (**a**) Schematic representation of VCE-004.8 synthesis. (**b**) VCE-004.8 and RGZ PPARγ binding affinities. The indicated concentrations were tested and results were plotted to obtain the polynomial trend on a logarithmic range. IC50 values are indicated within the graph (n = 3). (**c**) Effect of VCE-004.8 and HU-331 on PPARγ transcriptional activity. NIH-3T3 cells were co-transfected with GAL4-PPARγ and GAL4-luc. After 24 h, cells were incubated with the compounds at the indicated concentrations for 6 hours and lysed for luciferase activity. Results are expressed on a logarithmic scale as the fold induction ±S.D. relative to untreated control (n = 4). (**d**) Study of cytotoxic activity of VCE-004.8 and HU-331. NIH-3T3 cells were treated with the compounds at the indicated doses for 24 hours and cell viability was analyzed by the MTT method. Results are shown as mean ± S.D. and expressed as percentage of cell viability against untreated cells. (n = 3). (**e**) Binding affinity of VCE-004.8 to CB_2_ receptor. *Ki* values obtained from competition studies using [^3^H] CP55,940 as radioligand for hCB2 receptors and expressed as the mean ± SEM (n = 3). (**f**) VCE-004.8 is a CB_2_ receptor agonist. HEK-293T-CB_2_ cells were transfected with the plasmid pCRE-Luc containing three cAMP response elements and treated with WIN55212 or VCE-004.8 for 30 min and stimulated with FSK for 6 hours. Then, cells were lysed for luciferase activity. Data are shown as mean activation percentage ±S.D. considering FSK as 100% activation. (n = 6). *p < 0.05 **p < 0.01 ***p < 0.001 versus control ; ^#^p < 0.05 ^##^p < 0.01 versus FSK.

We have previously reported that the oxidation of CBG (Cannabigerol) and CBD to their corresponding quinols (VCE-003 and HU-331, respectively) increases the PPARγ binding activity of these natural cannabinoids^13^. HU-331 is a reactive quinone that shows potent antitumor activity by targeting DNA topoisomerase II^23^ and shows thiol trapping activity that induces oxidative stress and apoptosis in splenocytes^24^. Furthermore, HU-331 is rather unstable toward dimerization, that complete abolishes activity (unpublished data). In order to dissect the thiol-trapping activity of HU-311 from the PPARγ activity and stabilize the product toward oxidative dimerization, we capitalized on the electrophilicity of HU-331 to introduce a nitrogen function via a formal C-H functionalization. The resulting and fully substituted quinol (VCE-004.8, Fig. 1a) could not dimerize any more, and did not show any thiol trapping properties, as evidenced by a comparative cysteamine-recovery assay with HU-331. This cysteamine recovery assay was inspired by the cysteamine trapping assay^25^, a NMR method based on the treatment of a thiophylic compound with cysteamine in DMSO. This assay cannot be carry out on quinones due to the reversible formation of iminoquinones with the free amino group of the thiol probe that complicated the analysis of the spectra. A recovery assay was therefore developed, evaluating the recovery of the quinol from a solution of cysteamine in DMSO. While VCE-004.8 could be recovered unscathed in an essential quantitative way by extraction with hexane. In contrast HU-331 was undetectable in the hexane extract, suggesting that it had irreversibly reacted with cysteamine to form polar and not extractable adducts (see supplementary information for details).

Next, we first wanted to investigate whether VCE-004.8 was able to bind to PPARγ, and compare its binding capacity to RGZ. Using a PPARγ competitor-binding assay, we found that VCE-004.8 bounds to the nuclear receptor with an IC_50_ of 1.7 μM (Fig. 1b), significantly lower than the affinity comparatively evaluated for HU-331 (5 μM)^13^. To further study the ability of HU-331 and VCE-004.8 to activate PPARγ transcriptional activity, NIH-3T3 cells were transfected with a GAL4-PPARγ expression plasmid plus a GAL4-luc reporter plasmid. Our results showed that HU-331 induced PPARγ transactivation in a biphasic manner and this activity was lost with the higher concentrations tested. On the contrary, VCE-004.8 induced PPARγ transactivation in a concentration-dependent manner (Fig. 1c). The results obtained with HU-331 may reflect its cytotoxic activity and, therefore, NIH-3T3 cells were treated with increasing concentrations of both HU-331 and VCE-004.8 for 24 h. As depicted in Fig. 1d, HU-331 but not VCE-004.8 showed a clear cytotoxic activity with concentrations as low as 5 μM. In addition, we found that HU-311 induced reactive oxygen species (ROS), disrupted the mitochondrial transmembrane potential and activated the Nrf2 pathway, whereas none of these bioactivities were induced by VCE-004.8 (see Supplemenary Fig. S1). Altogether our results indicate that the specific modification introduced in the structure of VCE-004.8 did not affect its PPARγ binding activity but abolished its thiophilic and cytotoxic activity.

It is known that CBD does not bind directly to either CB_1_ or CB_2_ receptors but it can modulate CB_1_ activity^26^. Therefore, we were interested in investigating whether VCE-004.8 could interfere with these cannabinoid receptors. We studied the binding affinity of VCE-004.8 to CB_1_ and CB_2_ receptors and we found that VCE-004.8 could displace [^3^H]CP55,940 from specific binding sites in HEK-293T-hCB_2_ cell membranes with a *Ki* value of 170 ± 50 nM (Fig. 1e), with a negligible binding to CB_1_-containing membranes (*Ki* > 40 μM). Furthermore, we observed that VCE-004.8 repressed FSK-induced CRE-Luc activity in HEK-293T-CB_2_ cells, indicating that it is a functional CB_2_ agonist functional activity (Fig. 1f).

### VCE-004.8 inhibits collagen gen transcription and synthesis in fibroblasts

**Figure 2:**
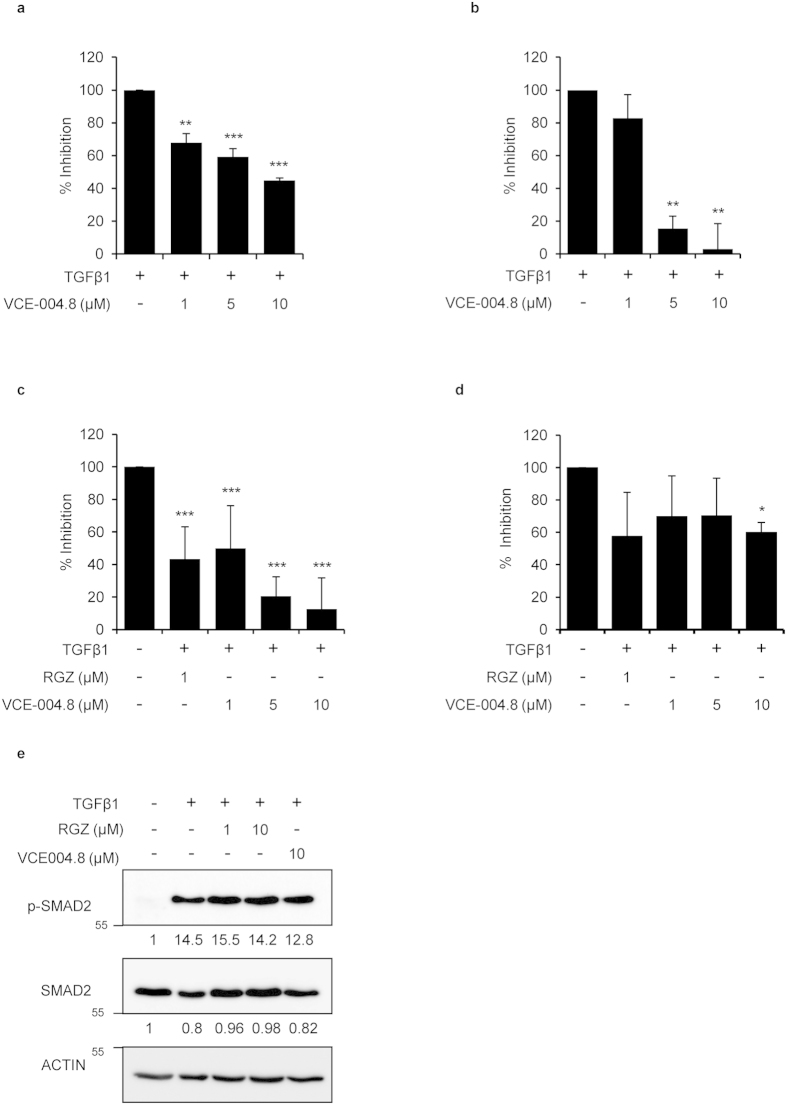
VCE-004.8 inhibits collagen deposition and gene transcriptional activation induced by TGFβ. NHDFs were preincubated with VCE-004.8 for 1 hour at the indicated concentrations and stimulated with TGFβ1 during 48 hours. (**a**) Cells were collected and tested for collagen content using Sirius Red-Fast Green method. (n = 5). (**b**) Supernatants were collected for procollagen type I measurement. Results are expressed as mean percentage of inhibition ±S.D. considering TGFβ1 as 100% (n = 3). (**c**) Effect of VCE-004.8 on Col1a2 transcriptional activity. NIH-3T3 cells were transfected with Col1a2-luc. Cells were preincubated with the compound at the indicated concentrations for 1 hour and stimulated with TGFβ1 (10 ng/ml) for 24 hours. Cells were lysed for luciferase activity. Results are expressed as mean percentage of inhibition ±S.D. considering TGFβ1 as 100% (n = 4). (**d**) Effect of VCE-004.8 on SMAD-dependent transcriptional activity. NIH-3T3 cells were transfected with the CAGA-Luc plasmid, preincubated with the indicated concentrations of VCE-004.8 for 1 hour and stimulated with TGFβ1 (10 ng/ml) for 6 hours. Then, cells were lysed for luciferase activity. Results are expressed as mean percentage of inhibition ±S.D. taking TGFβ1 as 100% (n = 4). (**e**) VCE-004.8 effect on SMAD2 phosphorylation. NIH-3T3 cells were incubated in low serum conditions (1% FBS) for 24 hours. Then, cells were pretreated with VCE-004.8 or RGZ for 1 hour and stimulated with TGFβ1 (10 ng/ml) for 2 hours. Protein expression was determined by Western Blot. Values under the gel indicate protein signal intensities after normalization to β-actin signal intensities. *p < 0.05 **p < 0.01 ***p < 0.001 versus TGFβ1 treated cells.

Since PPARγ agonists repress TGFβ-induced collagen gene transcription^11^, we evaluated if VCE-004.8 was also able to interfere with this pathway. We found that TGFβ-induced collagen deposition (46.4% induction vs control) and collagen synthesis (43.5% induction vs control) were significantly inhibited by pretreatment with VCE-004.8 (Fig. 2a,b, respectively). Type I collagen is a predominant ECM component of the fibrotic lesion, and is a product of two coordinately regulated genes, α1(I) (COL1A1) and α 2(I) (COL1A2). COL1A2 promoter has been extensively used as an experimental model system to delineate the transcriptional regulation of the collagen gene expression. Thus, to further demonstrate that VCE-004.8 inhibits collagen synthesis at the transcriptional level, NIH-3T3 cells were transiently transfected with the Col1A2-luc plasmid, containing the sequence from −376 to +58 of the human COL1A2 gene, and stimulated with TGFβ1 in the absence or presence of VCE-004.8 or RGZ. Fig. 2c shows that VCE-004.8 inhibited TGFβ-induced Col1A2 gene transcription in a concentration- dependent manner. As expected, RGZ also inhibited TGFβ-induced collagen gene transcription.

It has been shown that SMAD2/SMAD3 overexpression stimulates COL1A2 promoter activity^27^, and we therefore tested the capacity of VCE-004.8 to interfere with upstream or downstream TGFβ signaling pathways. We found that both compounds were able to inhibit the transcriptional activity driven by SMAD proteins in CAGA-Luc transfected NIH-3T3 cells (Fig. 2d). However, neither VCE-004.8 nor RGZ inhibited TGFβ-induced SMAD2 phosphorylation (Fig. 2e). These results are consistent with the view that PPARγ agonists inhibit the expression of several TGFβ-activated genes by acting at the transcriptional level.

### VCE-004.8 inhibits myofibroblasts differentiation and fibroblast migration

**Figure 3:**
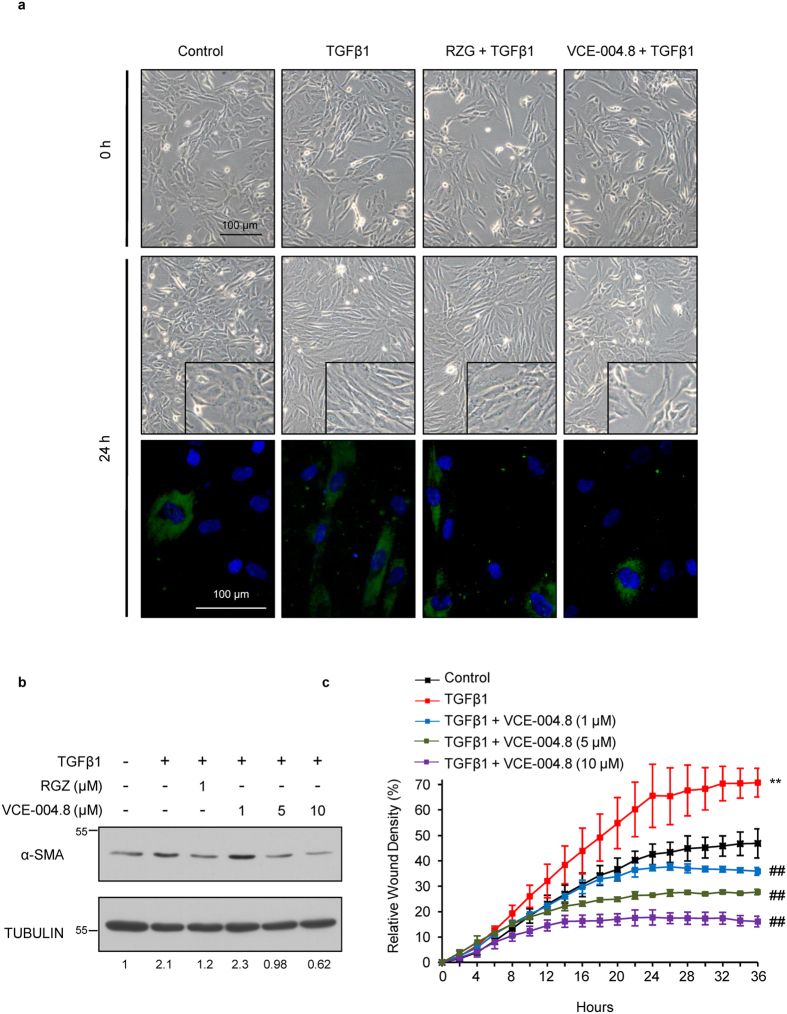
VCE-004.8 prevents differentiation into myofibroblast and impairs wound healing induced by TGFβ. (**a**) Representative images of MEFs differentiation into myofibroblasts. Cells were incubated in low serum conditions (1% FBS) for 24 hours. Then, cells were pretreated with VCE-004.8 for 1 hour and stimulated with TGFβ1 (10 ng/ml) for 24 hours. MEFs were stained for α-SMA (green) and DAPI (blue). Treatment with VCE-004.8 prevented TGFβ1 induction of α-SMA expression (n = 3). (**b**) VCE-004.8 reduces α-SMA protein expression induced by TGFβ. NIH-3T3 cells were incubated in low serum conditions (1% FBS) for 24 hours. Then, cells were pretreated with VCE-004.8 for 1 hour, stimulated with TGFβ1 (10 ng/ml) for 48 hours and the expression of α-SMA protein was determined by Western Blot. Values under the gel indicate α-SMA protein signal intensities after normalization to tubulin signal intensities and the results are representative of three independent experiments. (**c**) Scratch assay on NHDFs cells treated with either TGFβ in the absence or the presence of increasing concentrations of VCE-004.8. Results were plotted using the Incucyte^FLR^ software in terms of percentage of relative wound density ±S.D. as a function of time (n = 3). *p < 0.05 **p < 0.01 versus control; ^##^p < 0.01 versus TGFβ1 treated cells.

As previously described in the literature, SSc fibroblasts differentiate to myofibroblasts with prominent stress fibers and contractile properties that can be triggered by autocrine TGFβ activation (reviewed in^28^). Therefore, to study myofibroblastic differentiation *in vitro*, cells were labeled with antibody against α-SMA as specific marker for myofibroblasts. As shown in Fig. 3a, TGFβ1 induces α-SMA protein expression in cultured MEFs, which is associated with morphological changes of cellular hypertrophy, cell shape variations from stellate to bipolar and well-formed actin stress fibers, the hallmark of myofibroblast differentiation. Pretreatment with both RGZ and VCE-004.8 prevented cell morphology changes and decreased α-SMA expression as well as TGFβ1-induced cell proliferation detected by DAPI staining. We also found that RGZ and VCE-004.8 inhibited the steady state levels of α-SMA induced by TGFβ1 in NIH-3T3 cells (Fig. 3b).

Migration of skin fibroblast plays a crucial role not only in normal wound healing but also in SSc^29^. To further analyze this point, we tested if the anti-fibrotic effect of VCE-004.8 could be associated with reduced fibroblast migration. To investigate this possibility, monolayers of NHDFs cells were scratched and the effect of VCE-004.8 on TGFβ1-induced and rhIL-4-induced wound healing was analyzed during 36 h in the presence of mitomycin C. As is shown, VCE-004.8 significantly attenuated wound closure induced by either TGFβ1 or rhIL-4 in a concentration-dependent manner (Fig. 3c and supplementary Fig. 2 respectively).

### Effects of VCE-004.8 on fibrotic skin

**Figure 4:**
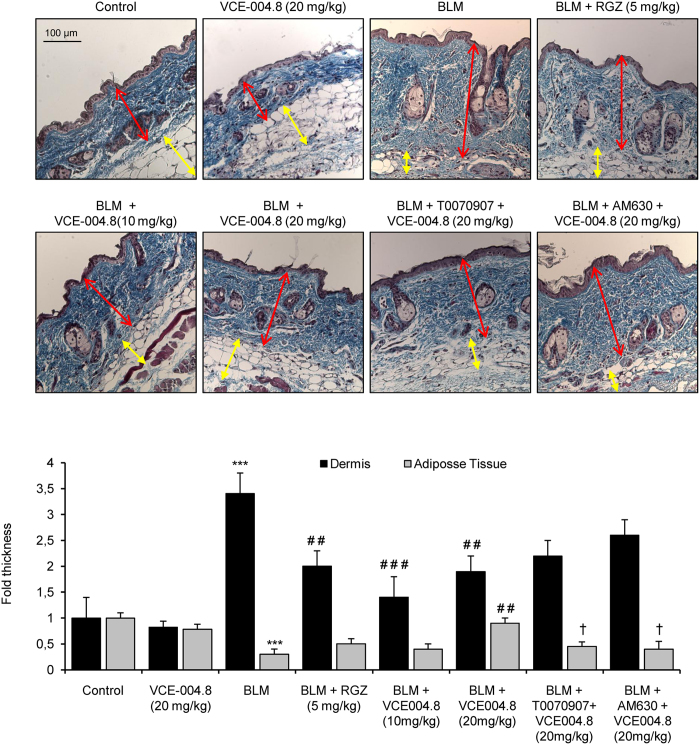
VCE-004.8 prevents dermal thickening induced by BLM administration. Images show Masson´s trichrome staining **(upper panel)** and their respective quantification **(bottom panel)** of skin from BLM-treated mice. Values are expressed as mean ± SEM (n = 8 animals *per* group). ^***^p < 0.001 versus control group; ^##^p < 0.01 ^###^p < 0.001 versus BLM group; and ^†^p < 0.05 versus BLM + VCE-004.8 (20 mg/kg).

**Figure 5:**
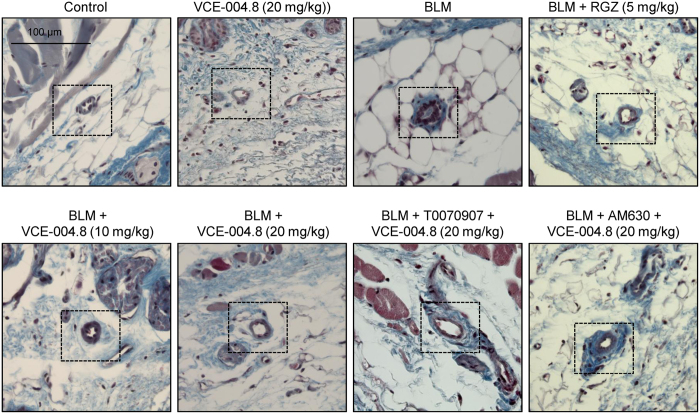
VCE-004.8 prevents BLM- induced collagen accumulation around blood vessels. Representative images of Masson’s trichrome stained skin sections showing collagen associated to blood vessels (indicated with frames). (n = 8 animals *per* group).

To study the anti-fibrotic effect of VCE-004.8 *in vivo,* we evaluated its effectiveness in a murine model of SSc. The induction was done by a 6 weeks regime of subcutaneous injections of bleomycin, followed by treatment with the different therapeutic regimen during the last 3 weeks. No signs of toxicity were observed during the experimental procedure. After 6 weeks, mice treated with bleomycin showed a significant increment in dermal thickness and collagen content that paralleled with a reduction of subcutaneous adipose layer, which was replaced by connective tissue. VCE-004.8 (10 and 20 mg/kg) alleviated skin fibrosis and reduced skin thickness to levels similar to those of RGZ. In addition, VCE-004.8 at the highest dose was also capable of recovering lipoatrophy. Pretreatment with either the CB_2_ antagonist AM630 or the PPARγ antagonist T007907 partially abrogated the effect of VCE-004.8, indicating that the anti-fibrotic response was dependent on dual PPARγ and CB_2_ activation (Fig. 4). Repeated local injections of bleomycin have been described to also lead to a significant thickness of vascular wall that mimicked some of histologic features found in human SSc^30^. As depicted in Fig. 5, bleomycin induced a significant collagen deposition around blood vessels that was prevented by both RGZ and VCE-004.8. Moreover, the effects of VCE-004.8 were abolished by pretreatment with the PPARγ and CB_2_ antagonists.

**Figure 6:**
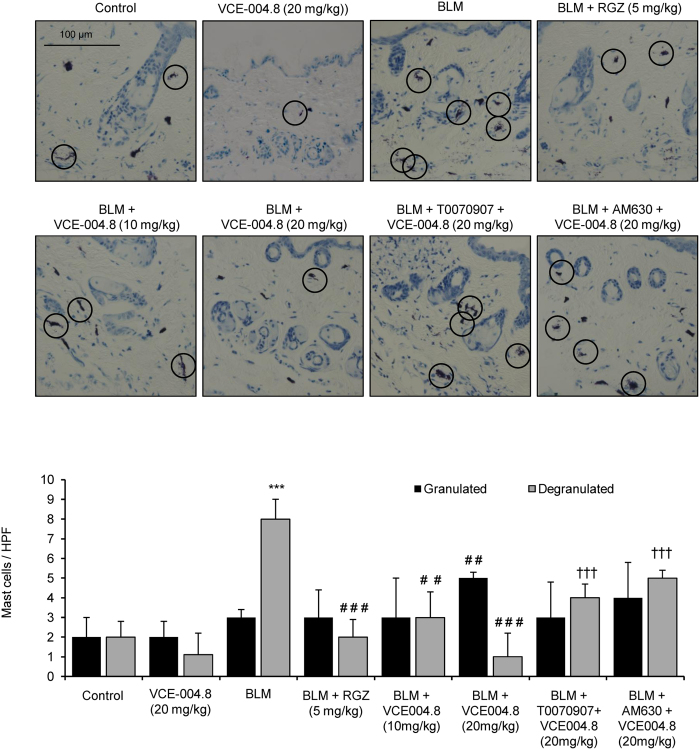
VCE-004.8 reduces BLM- induced mast cell degranulation in mice skin. **(Upper panel)** Toluidine blue staining of skin sections showing degranulated mast cells infiltration (indicated with circles). **(Bottom panel)** Quantification of toluidine labeled granulated/degranulated mast cells. Values are expressed as mean ± SEM (n = 8 animals *per* group). ^***^p < 0.001 versus control group; ^##^p < 0.01 ^###^p < 0.001 versus BLM group; and ^†††^p < 0.001 versus BLM + VCE-004.8 (20 mg/kg).

**Figure 7:**
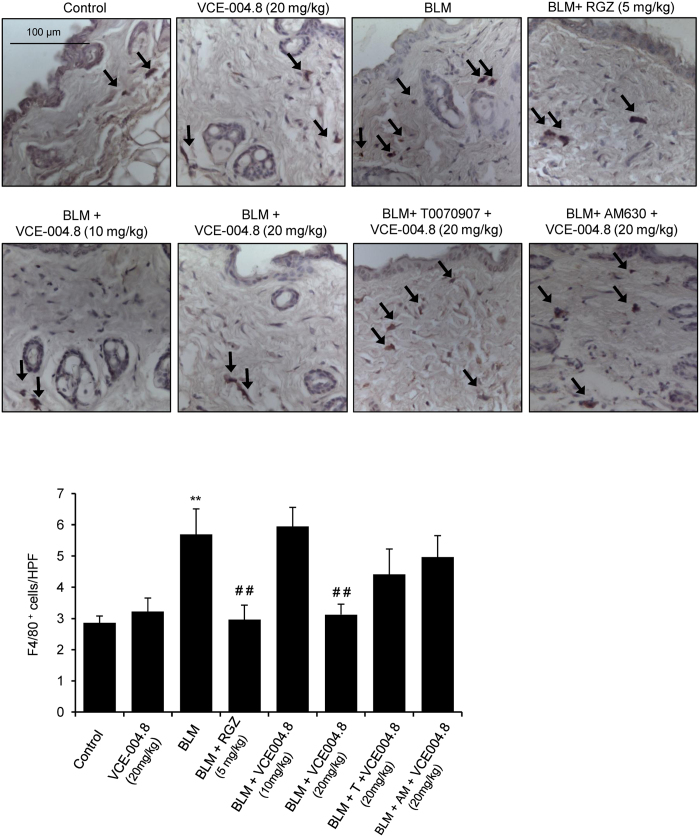
Treatment with VCE-004.8 reduces macrophage infiltration in the skin. **(Upper panel)** Images show immunostaining of skin sections for the macrophage specific marker F4/80 (indicated with arrows)**. (Bottom panel**) Quantification of F4/80(+) cells in skin. Values are expressed as mean ± SEM (n = 8 animals *per* group). ^**^p < 0.01 versus control group; ^##^p < 0.01 versus BLM group.

As previously demonstrated^31^, bleomycin-induced fibrosis is associated with changes in the number and function of mast cells and macrophage infiltration. Degranulating mast cells release profibrotic cytokines that induce collagen production^32^. Thus, skin mast cells were assessed for intact phenotype versus degranulating phenotype that was identified by the presence of extracellular granules. By staining with toluidine blue we found that bleomycin induced a significant increase of degranulated dermal mast cells that was significantly lessened in mice treated with RGZ or VCE-004.8 (10 and 20 mg/kg) (Fig. 6). Again, the effect of VCE-004.8 was partially blocked by pretreatment with PPARγ or CB_2_ antagonists. Next, we evaluated the effect of VCE-004.8 treatment on the recruitment of inflammatory cells by measuring the infiltration of F4/80(+) macrophages in the skin. When compared with control mice, bleomycin-treated skin samples showed a significant increase in the number of F4/80(+). Interestingly, VCE-004.8 (20 mg/kg), as well as RGZ, prevented macrophage infiltration in the skin. Although not statistically significant, mice pretreated with PPARγ or CB_2_ antagonist showed a higher number of F4/80 positive cells compared to VCE-004.8 (20 mg/kg) group (Fig. 7).

### VCE-004.8 and the expression of fibrotic-related genes in the skin of bleomycin-treated mice

**Figure 8:**
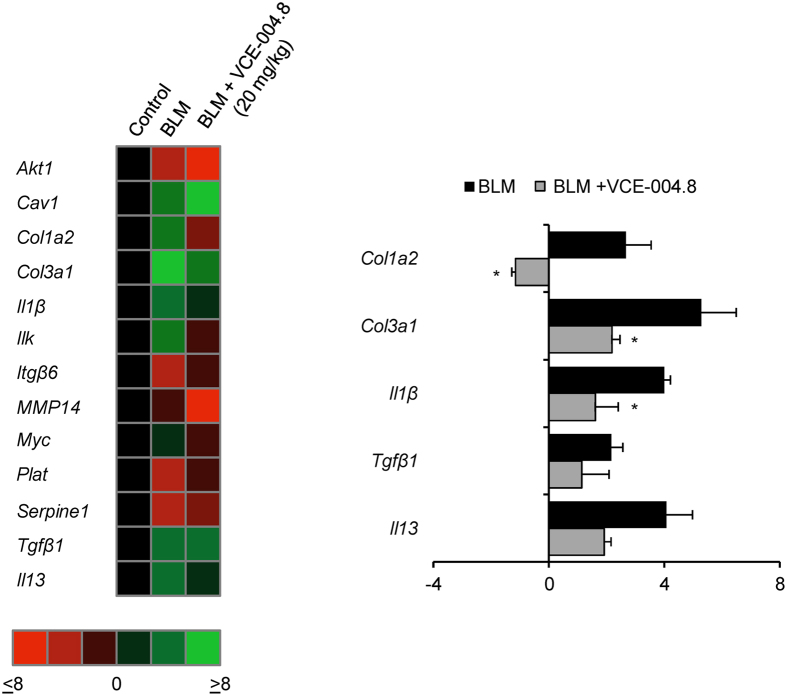
VCE-004.8 modulates gene expression in the skin of bleomycin-induced fibrosis in mice. Animals were treated as indicated and total RNA was isolated. 1 μg of RNA was retrotranscribed and the resulting cDNA was analyzed in a mouse fibrosis PCR array. **(Left panel)** Heat maps represents genes with significant upregulation (green) or downregulation (red) in the skin of BLM + vehicle or BLM + VCE004.8 (20 mg/kg) treated mice compared with control group. **(Right panel)** Those genes showing a relevant change in the expression pattern are shown and gene expression levels are expressed as fold up- or down-regulation during sickness (BLM + vehicle) and treatment (BLM + VCE-004.8 (20 mg/kg)). Five housekeeping genes contained on the experimental system were used to standardize the mRNA expression levels in every sample (n = 3). ^*^p < 0.05 versus BLM group.

In order to understand the molecular mechanisms underlying the beneficial effects of VCE-004.8 in bleomycin-induced skin fibrosis, mRNA was isolated from the skin of sick animals untreated and treated with VCE-004.8 (20 mg/kg), and the expression of 84 genes involved in fibrotic physiopathology was analyzed by qRT-PCR. The most striking results were obtained for the expression of *Col3A1*, *Col1A2*, *IL-1β* and *IL-13* genes, which were induced by bleomicyin and clearly inhibited by VCE-004.8 treatment (Fig. 8). In contrast, VCE-004.8 was not able to counteract the expression of *TGFβ1* and the induction of SMAD2 phosphorylation that were upregulated in the fibrotic tissue (supplemental Fig. 3). These results suggest that the activity of VCE-004.8 is related to the interaction with TGFβ1 signaling pathways downstream SMAD2 phosphorylation (i.e. *Col1A2* and *Col1A3* transcriptional inhibition) and by anti-inflammatory mechanisms (i.e. *IL-1β* and *IL-13* inhibition).

## Discussion

We have shown for the first time that VCE-004.8 is a dual PPARγ/CB_2_ agonist, devoid of affinity for the CB_1_ receptor and lacking thiophilicity, capable to inhibit collagen synthesis in fibroblasts and miofibroblast differentiation. In addition, this cannabinoid could efficiently prevent bleomycin-induced dermal fibrosis in mice.

Quinone based drugs exerting antibiotic and antineoplastic effects are commonly used in clinical practice, but their use for chronic treatments is not feasible because of its reactivity and toxicity. Thus, the development of cannabinoid quinonoid compounds displaying antiinflammatory activity that are more specific in their actions and are less toxic, is a major therapeutic goal. HU-311 shows increased PPARγ binding activity compared to its parent compound CBD^13^. However, HU-331 is also a thiol-trapping agent that induces ROS, disrupts the mitochondria transmembrane potential and induces cytotoxicity in primary and transformed cells *in vitro*. Here we report that VCE-004.8 is not a Michael-reactive compound as the electron-donating substituent eliminates thiophilicity by a combination of electronic (+M properties of nitrogen) and steric effects.

Thioglitazones such as RGZ and pioglitazone are potent PPARγ full agonists (PPARγ-fa) that have been largely used so far in the clinical practice. They provide similar effects on glycemic control, as well as a range of similar adverse effects, such as fluid retention, weight gain and increased risk of heart failure^33^. Indeed, RGZ was recently withdrawn in Europe and its use has been restricted in the USA as a consequence of increased risk of cardiovascular events in T2D patients. Beside the side effects of thioglitazones, PPARγ is still a major and relevant target to develop novel agonists that reduce or eliminate adverse effects for the management of difference diseases including SSc. Therefore, an intense research activity has developed for the discovery of selective PPARγ modulators (PPARγ-m) as safer alternatives to PPARγ-fa. In fact, RGZ is at least 20 fold more potent than VCE-004.8 to activate PPARγ. PPARγ-m only activate a subset of the functions induced by the cognate ligand or that act in a cell-type-selective manner^34^. In this sense VCE-004.8 can be considered a PPARγ-m based on the phytocannabinoid structural motif.

Blocking TGFβ signaling represents a therapeutic approach in fibrosis. PPARγ interferes with the SMADs transcriptional signaling without blocking its phosphorylation or nuclear accumulation^9^. Since PPARγ competes for the interaction of p300 to SMAD proteins inhibiting SMAD/p300 complex formation, which are required for TGFβ-induced collagen gene transcription^11^, it is conceivable that VCE-004.8 exerted most of their antifibrotic effects through PPARγ. However, CB_2_ activation may also be involved in the antiinflammatory activity of VCE-004.8 probably acting on macrophages inhibiting the expression of proinflammatory cytokines such as IL-1β. CB_2_ activation is thought to be beneficial in fibrotic diseases. For instance JWH-133, a CB_2_ selective agonist, has been shown to decrease the inflammatory infiltrate and fibrosis in cirrhotic rats^35^. It has also been described that CB_2_ activation inhibits dermal fibrosis by preventing leukocyte infiltration and the release of profibrotic mediators^19^. Thus, further research is warranted to investigate which antiinflammatory activities of VCE-004.8 are mediated by CB_2_ activation.

Experimental fibrosis induced by bleomycin administration is a widely used model to study SSc. Subcutaneous injections of bleomycin induce early inflammatory reaction followed by fibroblast activation and progressive fibrosis that resembling human disease^36^. We have used a modified model of bleomycin-induced fibrosis in which the treatment is initiated three weeks after the beginning of bleomycin injections in order to study the effect of VCE-004.8 on established fibrosis. This experimental model is less dependent of inflammation^14^,^37^. Consistent with previous studies, histological examination of the skin after bleomycin administration resulted in a significant dermal thickness and loss of subcutaneous adipose layer^30^. Treatment with VCE-004.8 during the last three weeks of bleomycin injections demonstrated a high inhibitory effect on the progression of dermal thickness and perivascular collagen deposition. In addition, VCE-004.8 was also effective when studied in a more inflammatory model of SSc that is induced by bleomycin administration during 3 weeks and treated in parallel with VCE-004.8 (data not shown). The infiltration of macrophages and degranulating mast cells, which represents an important source of TGFβ during fibrosis development^32^,^38^, was also reduced in mice receiving VCE-004.8. Although the levels of mRNA for TGFβ were not inhibited by VCE-004.8 in the tissues, it is possible that the inhibition of IL-1β and IL-13 mRNA expression observed in VCE-004.8-treated animals is reflecting a reduced macrophage infiltration and mast cell degranulation. Although mast cells are not necessary for inducing dermal fibrosis, they can produce IL-13, which together with other inflammatory cytokines such as IL-1β, may accelerate fibrosis development^39^,^40^. Overall, our results are in agreement with previous studies showing that PPARγ or CB_2_ agonists attenuated the infiltration of inflammatory cells in bleomycin-induced skin fibrosis^8^,^19^.

In conclusion, VCE-004.8 can be considered a PPARγ-m with CB_2_ agonistic activity that may qualify it as a candidate for the treatment of SSc and perhaps other fibrotic diseases whose management is currently elusive. The combination of dual agonists of PPARγ and CB_2_ receptors may represent an important advance in the development of novel therapies. In particular, it could be used for disorders with marked inflammatory profile, since both end-points are critical and complementary for the control of inflammatory pathways.

## Additional Information

**How to cite this article**: del Rio, C. *et al.* The cannabinoid quinol VCE-004.8 alleviates bleomycin-induced scleroderma and exerts potent antifibrotic effects through peroxisome proliferator-activated receptor-γ and CB2 pathways. *Sci. Rep.* **6**, 21703; doi: 10.1038/srep21703 (2016).

## Supplementary Material

Supplementary Information
