# Supplementary material for: The cannabinoid quinol VCE-004.8 alleviates bleomycin-induced scleroderma and exerts potent antifibrotic effects through peroxisome proliferator-activated receptor-γ and CB2 pathways
Source: Sci Rep. 2016 Feb 18;6:21703. doi: 10.1038/srep21703 (PMC4757881; doi:10.1038/srep21703)
Supplement: Supplementary Information [file srep21703-s1.docx]

**Supplemental information**

**Title:** The cannabinoid quinol VCE-004.8 alleviates bleomycin-induced scleroderma and exerts potent antifibrotic effects through peroxisome proliferator-activated receptor-γ and CB2 pathways.

**Authors:** Carmen del Rio^1^, Carmen Navarrete^2^, Juan A. Collado^2^, M. Luz Bellido^2^, Maria Gómez-Cañas^3-5^, M. Ruth Pazos^3-5^, Javier Fernández-Ruiz^3-5^, Federica Pollastro^6^, Giovanni Appendino^6^, Marco A. Calzado^1^, Irene Cantarero^1*^ and Eduardo Muñoz^1*^

**VCE-004.8 synthesis.** Benzylamine (1.3 mL, 11.913 mmol) was added to a solution of HU-331 (117 mg, 0.303 mmol) in EtOH (13 mL). The reaction mixture was stirred at r.t. for 18 h. It was poured into H_2_O (50 mL), taken up to pH=2 with HCl (10% aqueous solution) and extracted with CH_2_Cl_2_ (30 mL). The organic layer was dried over Na_2_SO_4_ (anhydrous), filtered and concentrated. Crude residue was purified by reverse phase chromatography (30/70% vol/vol CH_3_CN/H_2_O) to give 87 mg of (1'R, 6’R)-3-(benzylamino)-6-hydroxy-3'-methyl-4-pentyl-6'-(prop-1-en-2-yl)-[1,1'bi(cyclohexane)]- 2',3,6-triene-2,5-dione [purple-colored solid, yield: 66%].

**^1^H NMR** (CDCl_3_, 300 MHz) d ppm: 8.30 (bs, 1H), 7.44-7.26 (m, 5H), 6.64 (m, 1H), 5.15 (s, 1H), 4.65 (d, *J* = 6.0 Hz, 2H), 4.59 (m, 2H), 3.64 (m, 1H), 2.73 (m, 1H), 2.47 (t, *J* = 7.7 Hz, 2H), 2.30-1.76 (m, 4H), 1.68 (s, 3H), 1.64 (s, 3H), 1.54-1.23 (m, 6H), 0.88 (m, 3H)

**Cysteine recovery assay.** 10 mg of HU-331 and a molar equivalent amount of VCE-004.8 were independently dissolved in 1 mL DMSO, and each solution was next treated with an excess (4 mol. equivalents) of cysteamine. After stirring at room temperature for 1 h, the solutions were diluted with water (2 mL) and extracted with hexane- ether 9:1. After evaporation, the solution was taken up in CDCl_3_ and analyzed by ^1^H-NMR. While compound VCE-004.8 could be recovered unscathed in an essential quantitative way, HU-331 was undetectable in the residue, indicating that it had irreversibly reactive with cysteamine to form polar and not extractable adducts.

**Determination of ROS.** Jurkat cells were seeded at 10^6^ cells/ml in 24-well plates and treated with VCE004.8 for 6 hours. Then, the cells were stained with 5-(and-6)-chloromethyl-2′,7′-dichlorodihydrofluorescein diacetate acetyl ester (CM-H_2_DCFDA; Invitrogen) at 1 μM for 20 minutes at 37°C,. Later, washed twice with cold-PBS and maintained on ice until analysis. Cells exposed to pro-oxidant tert-Butyl hydroperoxide (TBHP) were used as positive control. Oxidation of H_2_DCFDA was detected by flow cytometry using a BD FACSCanto^TM^ (BD Bioscience, CA, USA).

**Determination of mitochondria transmembrane potential.** Jurkat cells were seeded at 10^6^ cells/ml in 24-well plates and incubated for 2 hour with VCE004.8, HU331 or *tert*-Butyl hydroperoxide (TBHP) as a positive control for loss of mitochondrial membrane potential. After that the cells were washed twice with cold PSB and stained with MitoTracker® Red CMXRos (Life technologies) for 20 min at 37ºC in the darkness. After washing with PBS the cells were analyzed by flow cytometry

**Activation of the Nrf2 pathway.** HaCaT cells stably expressing ARE-Luc reporter were seeded at 5x10^3^ cells/well in 96-well plates. Then, cells were treated with increasing concentrations of VCE-004.8 or *tert*-Butyl hydroquinone (TBHQ) as positive control for 24 hours. After stimulation the luciferase activities were quantified using Dual-Luciferase Assay.


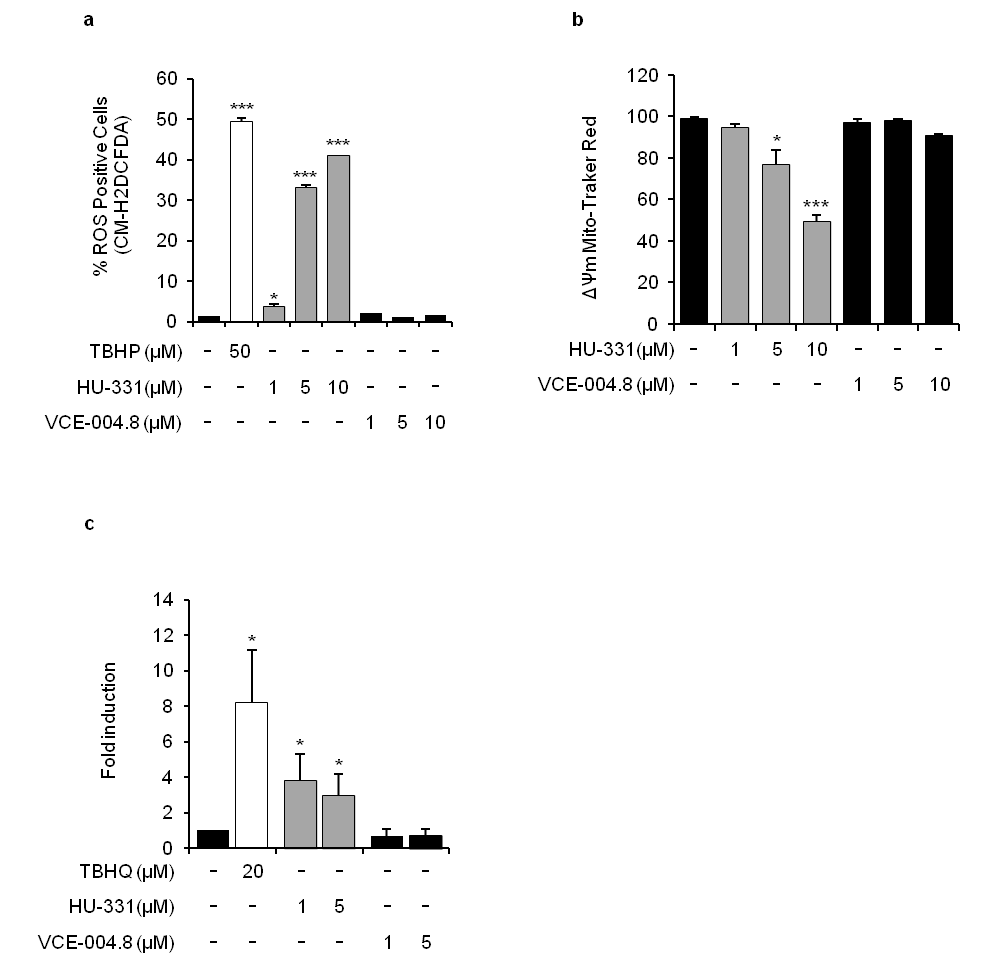


**Supplementary Figure S1. VCE-004.8 is a non-electrophilic compound. (a)** Reactive oxygen species production. Jurkat cells were treated with VCE004.8 or HU-331 for 6 hours. Then, cells were stained with CM-H_2_DCFDA and measured by flow cytometry. Results are expressed as mean percentage of ROS positive cells ± S.D. **(b)** Effect on mitochondrial membrane potential. Jurkat cells were treated with VCE004.8 or HU-331 for 2 hours. Then, cells were stained with MitoTracker® Red CMXRos and measured by flow cytometry. Results are expressed as mean percentage ± S.D**. (c)** Effects on Nrf2 activation**.** ARE-Luc HaCat cells were incubated for 6 h with VCE-004.8 or HU-331 at the indicated concentrations and lysed for luciferase activity. Results are expressed as mean fold induction ± S.D relative to control sample. All the results are representative of at least three independent experiments.


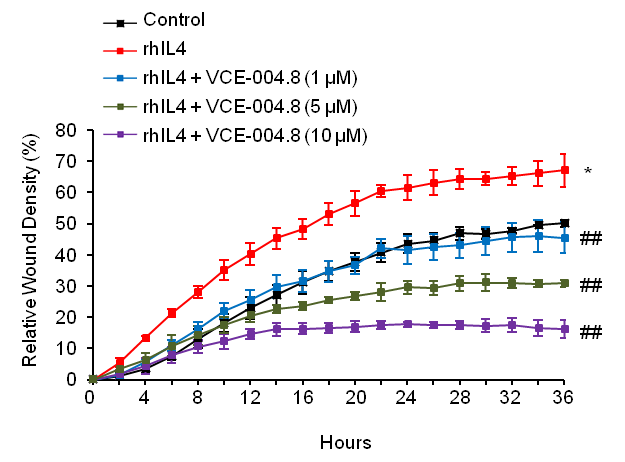


**Supplementary Figure S2.** Scratch assay on NHDFs cells treated with rhIL-4 in the absence or the presence of increasing concentrations of VCE-004.8. Results were plotted using the Incucyte^FLR^ software in terms of percentage of relative wound density ± S.D. as a function of time and are representative of two independent experiments performed in triplicate wells. ^*^p<0.05 ^**^ p<0.01 versus control; ^##^ p<0.01 versus rhIL4 treated cells.


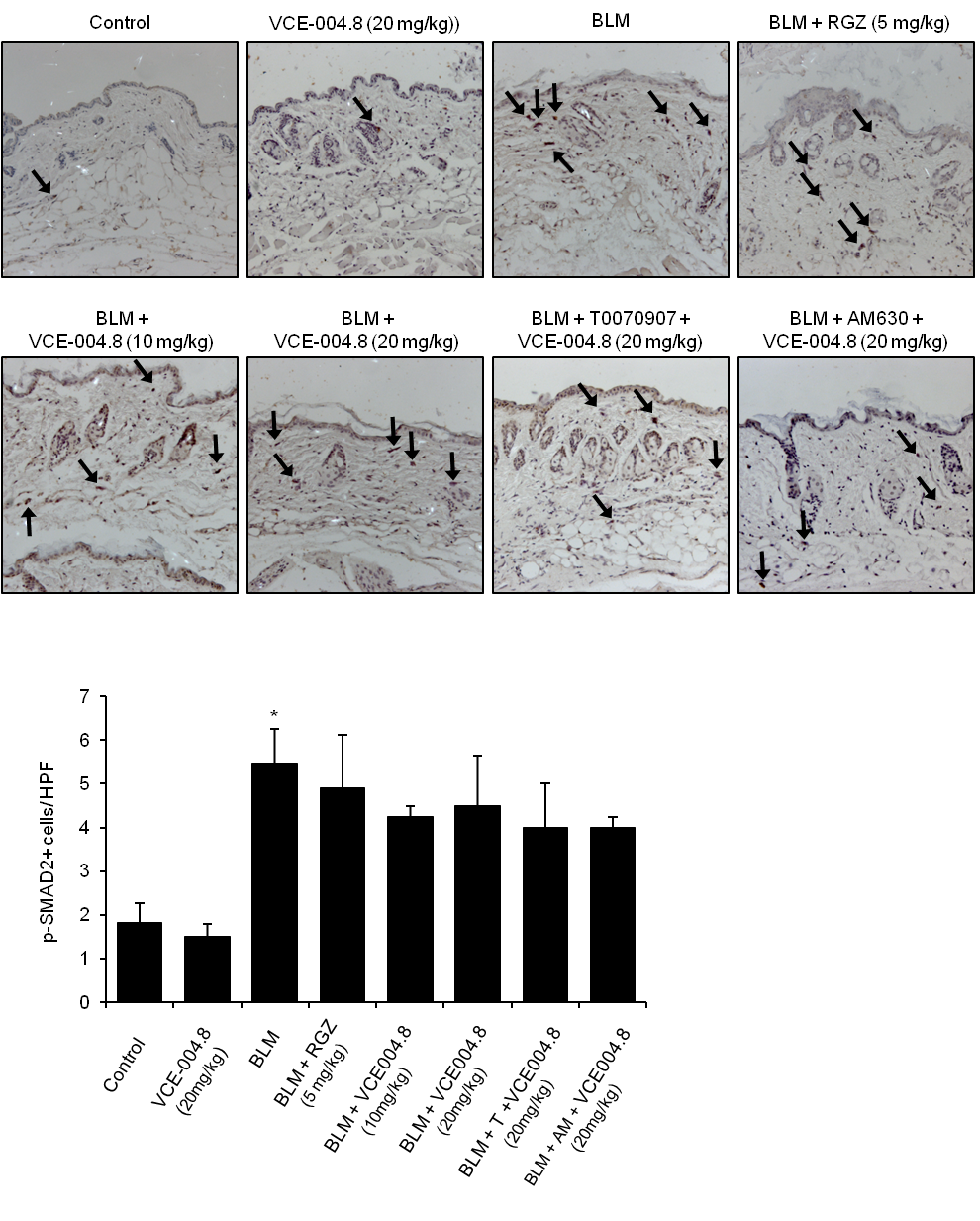


**Supplementary Figure S3. Effect of VCE-004.8 on SMAD2 phosphorylation in the skin. (Upper panel)** Images show immunostaining of skin sections for p-SMAD2 (indicated with arrows)**. (Bottom panel**) Quantification of p-SMAD2(+) cells in skin. Values are expressed as mean ± SEM (n=8 animals *per* group). ^*^ p<0.05 versus control group.
